# Interrelationships Among Childhood Trauma, Insecure Attachment, Dissociation, Negative Schemas and Psychosis Symptoms: A Network Analysis

**DOI:** 10.1002/cpp.70187

**Published:** 2025-12-12

**Authors:** David Levi, Mathias Pierce, Alison Baird, Katherine Berry

**Affiliations:** ^1^ School of Health Sciences, Faculty of Biology, Medicine and Health, Manchester Academic Health Sciences Centre The University of Manchester Manchester UK; ^2^ Research and Innovation Greater Manchester Mental Health NHS Foundation Trust Manchester UK

## Abstract

**Background:**

Childhood trauma is associated with increased rates of psychosis, with research identifying insecure attachment, dissociation and negative schemas as mediating factors. Network analysis offers a framework to explore complex interrelationships between symptoms and vulnerability factors. This study used network analysis to examine connections between experiences of childhood trauma, insecure attachment, dissociation, negative schemas and positive and negative psychosis symptoms. The study aims were to identify central, strongly connected variables and to compare network structures between subgroups with (*n* = 259) and without (*n* = 350) a self‐reported psychosis diagnosis.

**Methods:**

Cross‐sectional, self‐report data from an online survey sample (*N* = 609) were analysed. The sample was predominantly male (72.5%), White British/Other (80.1%), with a mean age of 28.70. The psychosis diagnosis sample contained more females (30.1%) than the sample without a psychosis diagnosis (21.1%). The psychosis diagnosis sample was also older, with a mean age of 32.93 compared with the no psychosis diagnosis sample (25.56). Pairwise Markov random field (PMRF) networks and strength centrality indices were used to identify central variables, while a network comparison test (NCT) evaluated structural differences between subgroups.

**Results:**

Bizarre experiences showed strong connectivity with other positive symptoms. Negative‐self schemas and paranoia demonstrated strong cross‐construct connectivity, forming a cluster with negative‐other schemas, avolition and insecure attachment. Depersonalisation–derealisation was strongly connected to hallucinations. The NCT revealed no significant structural differences between subgroups.

**Conclusions:**

Targeting negative‐self schemas and paranoia may yield the most substantial reductions in cross‐construct network connectivity and are indicated for treatment prioritisation. Interventions targeting detachment‐related dissociation may help reduce hallucinations. Longitudinal studies are needed to replicate these findings and explore causal pathways within network structures.

## Summary

- Paranoia and avolition symptoms were implicated in a cluster of strong, potentially reinforcing associations with anxious attachment and negative‐self and other schemas. Interventions that weaken these links (e.g., reducing threat‐based thinking and safety behaviours such as avoiding social contact) may yield greater clinical gains than addressing these symptoms in isolation.
- Detachment‐type dissociation appears central to hallucinations. Techniques such as grounding, distress tolerance and mindfulness may help clients predict, control and reduce dissociative episodes, thereby reducing hallucinatory experiences.
- Use of multidimensional measures of psychological processes linked to psychosis symptoms (e.g., schema and insecure attachment subtypes and dissociation domains) provide richer information for formulations that can help identify and target reinforcing psychopathology more effectively.

## 1 Introduction

Childhood trauma increases vulnerability to developing psychosis (Read et al. 2005; Varese et al. 2012). Numerous mediating factors have been implicated in the pathway from childhood trauma to psychosis, including insecure attachment (Carr et al. 2018; Gumley et al. 2014), negative schemas (Alameda et al. 2020) and dissociation (Longden et al. 2020). The current study explores the complex interrelationships between these mediating factors, childhood trauma and psychosis symptoms.

Insecure attachment refers to patterns of relational insecurity that develop in the context of disrupted or unresponsive early caregiver relationships and influence relational functioning in adulthood (Bowlby 1969). Three predominant adult insecure attachment styles have been conceptualised: (1) anxious attachment, characterised by negative‐self beliefs, high expressed emotion and seeking proximity to others, combined with fears of rejection; (2) avoidant attachment, characterised by negative views of others and seeking to maintain relational distance; and (3) disorganised attachment, characterised by fluctuations between anxious and avoidant strategies (Bartholomew 1994; Mikulincer and Shaver 2010). Different attachment styles, with their varying behavioural and affect‐regulation characteristics, may have divergent relationships with specific symptoms and other associated processes. Identifying these divergent relationships could elucidate the behavioural and affect‐regulation characteristics most implicated in specific psychosis symptoms, thereby supporting hypothesis generation on intervention targets.

Dissociation has been implicated in the development of hallucinations but also shows robust associations with paranoia (Longden et al. 2020). Dissociation has been conceptualised as a multidimensional construct involving ‘detachment’ (e.g., depersonalisation–derealisation) and ‘compartmentalisation’ (e.g., dissociative amnesia) phenomena and nonpathological experiences such as ‘absorption’ (Brown 2006; Holmes et al. 2005). The associations between specific psychosis symptoms and multidimensional aspects of dissociation warrant further investigation (Berry et al. 2017).

While insecure attachment focuses on the behavioural and affective components of relational functioning, negative schemas focus on explicit cognitive architecture thought to underpin negative‐self/other representations (Karantzas et al. 2023). A growing body of evidence suggests that insecure attachment styles and negative schemas are associated with psychosis symptoms and may have particularly influential roles in the development of paranoia (Humphrey et al. 2021; Lavin et al. 2020). Whether this ‘special relationship’ holds within a network of interconnections involving constructs such as dissociation, also linked to paranoia (Longden et al. 2020), has not been tested to date.

Much less is known about the factors contributing to the development and maintenance of negative symptoms (Lyne et al. 2017). Some research highlights potential influences of negative schemas (Grant and Beck 2009; Jaya et al. 2017), stigma (Ordóñez‐Camblor et al. 2021) and attachment avoidance in non‐clinical populations (Carr et al. 2018); however, a coherent model for the development and maintenance of negative symptoms is currently lacking.

Psychosis research has tended to focus on the aetiology of specific positive symptoms in isolation or on summary measures of ‘positive’ and ‘negative’ symptoms. Less is known about the interrelationships between the multidimensional aspects of symptoms and their proposed causal factors. Network analysis has emerged as an alternative approach, positing that mental health difficulties are best described as systems of complex associations between symptoms and biopsychosocial factors, all of which have discreet causal power to influence one another (Borsboom 2017; Borsboom and Cramer 2013). Network models graphically depict psychological symptoms and other relevant psychological processes as nodes (points in space) and their interassociations as edges (thicker lines/edges representing stronger associations). Clusters of strong associations represent ‘activated’ networks in which pathological processes are thought to become self‐sustaining, thus constituting ‘mental disorder’ (Borsboom 2017). Statistical analyses can identify the most central nodes within the network (those with the most/strongest connections), thereby facilitating inferences on the most influential factors/combination of factors implicated in network activation.

Much research into psychological mechanisms involved in the development and maintenance of psychosis symptoms occurs within non‐clinical populations. The efficacy of this research relies in part on the assumption that psychotic symptoms are not the preserve of a distinct clinical population but rather, lie on a continuum of severity (e.g., Strauss 1969) and are observed across clinical and non‐clinical populations (Claridge 1972; Meehl 1962, 1989). Research into the psychosis continuum has tended to focus on the nature and frequency of psychosis symptoms across clinical and non‐clinical populations, rather than the structure of associations between symptoms and proposed causal factors (DeRosse and Karlsgodt 2015). A useful advance in this field would be to understand whether the structure of complex associations between symptoms and proposed causal variables shows continuity across clinical and non‐clinical samples.

Previous network analyses have explored phenomena related to those described above. A ‘shortest path’ analysis used to investigate network paths between childhood trauma and psychosis symptoms through ‘general psychopathology’ found that childhood trauma was not directly associated with any symptoms and only connected through general psychopathology variables (Isvoranu et al. 2017). Additionally, paranoia, a common feature of psychosis, was found to have an important bridging role, connecting adverse neighbourhood social environment factors and depression and anxiety factors, but only in conditions of high social deprivation (McElroy et al. 2019). A study by Brasso et al. (2023) investigated differences in network structures of symptoms, cognition and real‐life functioning between early and late‐phase schizophrenia samples. They found no significant difference in the overall network structure, indicating network structures may remain stable over illness duration. Černis, Evans, et al. (2021) and Schlesselmann et al. (2023) used network analysis to examine relationships between dissociative and psychosis symptoms. Černis, Evans, et al. found dissociation was connected to paranoia, grandiosity and cognitive disorganisation. Schlesselmann et al. showed dissociative symptoms in schizophrenia‐spectrum disorders clustered separately from psychosis but were indirectly linked via emotional distress. Other network studies within the psychosis literature have used repeated‐measures designs (e.g., Kuipers et al. 2019; Moffa et al. 2023) to identify ‘causal cascades’ between psychosis symptoms and putative causal factors including adverse experiences, nonpsychotic affective difficulties (e.g., generalised anxiety and depressed mood) and substance use. While repeated‐measures data are optimal in exploring causal relationships and give credence to the use of longitudinal network analytic methods such as directed acyclic graphs, it is resource intensive. It is hoped the current study, and its use of cross‐sectional data, will highlight important relationships worthy of future longitudinal research.

This study aimed to assess the interrelationships between multidimensional measures of childhood trauma, insecure attachment, dissociation, negative schemas and specific positive and negative symptoms, within a large online sample of clinical and non‐clinical participants. The study had a further aim of assessing whether the structure of networks differed significantly for a subsample of participants reporting a diagnosis of psychosis compared with those reporting no psychosis diagnosis. The specific study aims were as follows:

- Identify variables with the highest ‘centrality’ (influence) within the network, thereby providing insights into the symptoms and related psychological factors most influential in activating other nodes within the network.
- Identify variables with the strongest between‐construct (‘bridging’) connections. It was anticipated there would be high levels of intraconstruct connectivity (e.g., positive symptom–positive symptom associations and dimensions of dissociation being strongly associated). This aim hoped to provide insights into the literature‐proposed causal/mediating factors with the strongest connectivity within the network, ‘above and beyond’ their intraconstruct connectivity.
- Identify clusters of strong network connections, and discuss their relevance to previous research, and implications for future research and clinical practice. In accordance with the network theory of mental health disorders (Borsboom 2017), this would provide insights into which symptoms and associated processes under investigation are most influential in forming self‐sustaining negative feedback loops, characteristic of mental disorder.
- Identify if the structure of two networks for individuals with or without a self‐reported diagnosis of psychosis differed significantly. This aim intended to advance the debate on the continuum theory of psychosis by assessing whether the structure of associations between psychosis symptoms and relevant psychopathological processes demonstrated continuity between those with a self‐reported psychosis diagnosis and those without.

## 2 Method

### 2.1 Participants

Data were collected through online surveys as part of an ongoing programme of research at the University of Manchester, providing a total sample of *n =* 609. Study advertisements were shared online via social media and via the University student research portal. Mental health services and charitable psychosis support networks were also contacted to share the study advert. Participants were invited to take part whether or not they reported a diagnosis of psychosis or having received treatment in relation to psychotic experiences. Participants needed to be at least 18 years old and to be proficient in English to complete the survey. All responses were self‐report, and there was no triangulation of health records or clinician oversight regarding the diagnostic information provided. Analyses on a subset of the data (*n =* 242) have been published in two previous studies (Humphrey et al. 2022; Degnan et al. 2022). All data included in this study were collected through protocols approved by the local ethics committee (UREC Ref: 2019‐5562‐9487). All further procedures and analyses were performed according to the ethical standards formulated by this committee, and no further ethical approval was required for the current secondary analysis study.

### 2.2 Measures

A screening questionnaire collected information such as gender, ethnicity, employment, educational attainment and clinical information such as diagnoses.

The Brief Betrayal Trauma Survey (BBTS: Goldberg and Freyd 2006) is a 12‐item self‐report measure of traumatic experiences, before and after the age of 18. Respondents rate their responses on a 3‐point Likert scale. Higher scores indicate more exposure to traumatic events. The BBTS has demonstrated good construct validity and test–retest reliability (Goldberg and Freyd 2006). Considering the evidence of associations between specific forms of abuse and specific psychotic symptoms (Bentall et al. 2014), specific items relating to sexual (two items), physical (two items) and emotional (one item) abuse were used in the current study. Response data for the emotional abuse item were transformed to a binary categorical variable (present/not present) for analysis.

The Psychosis Attachment Measure—Revised (PAM‐R) (Pollard et al. 2020) is a 23‐item self‐report questionnaire of insecure attachment in individuals with psychosis. Respondents rate their endorsement of items on a 4‐point Likert scale. Scores are yielded for anxious, avoidant and disorganised attachment, with higher scores indicating stronger endorsement of the associated insecure attachment style. Internal reliability for all three subscales in the current sample was good (*α* > 0.80).

The Dissociative Experiences Scale—Revised (DES) (Carlson and Putnam 1993) is a 28‐item self‐report yielding three subscales: depersonalisation–derealisation (detachment), amnesia and absorption. Items are summed and divided by the number of items to produce a subscale total score. Higher scores denote increased dissociative experiences. Internal reliability for all three subscales in the current study was good (*α* > 0.80).

Negative‐self/other schemas were assessed using the Brief Core Schema Scales (BCSS) (Fowler et al. 2006). The BCSS was designed for use in psychosis populations and contains 24 items assessing core beliefs about the self and others. Responses are rated on a 5‐point Likert scale and higher scores denote higher agreement. Subscale total scores are obtained for (1) negative‐self, (2) positive‐self, (3) negative‐other and (4) positive‐other. Only the negative subscales were utilised in the current study. Internal consistency statistics for the negative schema subscales in the current study were good for negative‐self (*α* = 0.88) and excellent for negative‐other schemas (*α* = 0.91).

The Community Assessment of Psychic Experiences (CAPE) (Stefanis et al. 2002) is a 42‐item measure assessing the frequency and distress of positive, negative and depressive psychosis‐like symptoms. Respondents rate their endorsement of items on a 4‐point Likert scale with higher scores denoting higher symptom frequency/distress. It has demonstrated good psychometric properties within clinical and non‐clinical studies (Konings et al. 2006; Stefanis et al. 2002). Frequency scores for specific positive and negative symptom domains were calculated based on a previous factor analysis (Schlier et al. 2015). These included positive symptom domains for (1) hallucinations, (2) paranoia, (3) bizarre experiences, (4) grandiosity and (5) magical thinking and negative symptom domains for (1) social withdrawal, (2) affect flattening and (3) avolition. The internal reliability for symptom‐specific subscales was good for all domains (*α* > 0.80) other than grandiosity (*α* = 0.77) and social withdrawal (*α* = 0.74) which were acceptable and magical thinking (*α* = 0.63) which was questionable.

## 3 Statistical Analyses

Preliminary data screening was completed on SPSS (Version 25). Data imputation and network analyses were completed on R (Version 4.2.2). Missing data on measurement variables across the entire sample ranged between 9.8% and 22%. Chi‐square goodness‐of‐fit tests showed disproportionate amounts of missing data on several variables when grouped by key demographics including sexuality, educational attainment and psychosis diagnosis. Missing data were therefore imputed using the missForest package within R (Stekhoven and Bühlmann 2011), which uses an iterative imputation method based on random forests suitable for non‐parametric, mixed‐type data. Observations of histograms and Kolmogorov–Smirnov tests showed that all continuous variables were non‐normally distributed, a majority presenting with positive skew. To relax normality assumptions, nonparanormal transformation was applied to continuous variables using the R Huge package (Liu et al. 2012).

Three networks were calculated and depicted, one for the overall sample and two further networks for the psychosis diagnosis/no psychosis diagnosis subgroups. For each ‘main’ network, an accompanying ‘bridge edges only’ (BEO) network graph was produced, showing only non‐zero edges between constructs, setting intraconstruct edges to zero.

The pairwise Markov random field (PMRF) class of statistical model was used. Within these networks, variables are represented as ‘nodes’, while the associations between nodes are represented as ‘edges’. Edges represent unique associations between two nodes, controlling for all other associations. The relative importance of nodes in defining the structure of the network is measured through their ‘centrality’—those with the most and strongest edges featuring more centrally within the network. Strength, closeness and betweenness centrality indices are often reported in psychological networks. However, there are considerable theoretical challenges with applying closeness and betweenness centrality and associated inferences of ‘symptom spread’ to cross‐sectional, psychological networks (Bringmann et al. 2019). Therefore, the current study only reported on strength centrality indices. Centrality indices are reported as standardised *z* scores and higher values indicate greater importance to, and influence within, the network.

The stability of network structures was evaluated with non‐parametric bootstrapping procedures. Dropping of cases with 1000 bootstrap samples (see Data S1 for further details) assessed the proneness of centrality indices and edge weights to sampling variation (Bringmann et al. 2019).

Due to the inclusion of continuous and categorical variables, the ‘mgm’ function within the R bootnet package was used to estimate mixed graphical networks (Haslbeck and Waldorp 2020). The least absolute shrinkage and selection operator (LASSO) was employed along with the extended Bayesian information criterion (EBIC) hyperparameter set to the default 0.5. The Fruchterman–Reingold algorithm ‘spring’ graph was used to visualise network graphs (Fruchterman and Reingold 1991), alongside a ‘circle’ layout highlighting bridging (interconstruct edges) connections.

Comparisons of the psychosis diagnosis/no psychosis diagnosis subgroup networks were conducted using the network comparison test (NCT) R package (van Borkulo et al. 2023). Global network structure and strength for the two groups were compared using the M‐test (structure) and S‐test (global strength). The between‐group comparison of specific edge strengths was conducted using the edge invariance test, applying the Holm–Bonferroni correction for multiple comparisons with significance set at *p* < 0.05.

## 4 Results

### 4.1 Descriptive Statistics

The study sample included 72.5% males, 25% females and 2.5% preferred to self‐describe their gender. Socio‐demographic and clinical characteristics for the entire sample (*n* = 609), psychosis diagnosis (*n =* 259) and no psychosis diagnosis (*n* = 350), subsamples are presented in Table 1. *t* tests and chi‐square goodness‐of‐fit statistics found the variables of age, gender, ethnicity, educational attainment and employment to be disproportionately spread across the psychosis/no psychosis diagnosis groups. Participants in the psychosis diagnosis group were disproportionately more likely to be older, female or prefer to self‐describe, be of ‘White other’ ethnicity, unemployed or in receipt of benefits and not have attained degree‐level qualifications. Table 2 presents study measure characteristics for the same groups. The psychosis diagnosis group reported statistically significant higher scores on all measures (all *p* < 0.001).

**TABLE 1:** Descriptive statistics for socio‐demographic characteristics for the entire sample, psychosis diagnosis and no psychosis diagnosis groups.

	Entire sample *n =* 609 [%] (SD)	Psychosis diagnosis *n* = 259 [%] (SD)	No psychosis diagnosis *n* = 350 [%] (SD)
Gender			
Males	441 [72.5]	168 [64.9]	273 [78.2]
Females	152 [25.0]	78 [30.1]	74 [21.1]
Prefer to self‐describe	15 [2.5]	13 [5.0]	2 [0.6]
Missing (other data provided)	1 [0.2]	0	1 [0.3]
Age	28.70 (11.52)	32.93 (13.31)	25.56 (8.76)
Ethnicity			
White British	360 [59.1]	129 [49.8]	231 [66.0]
White other (European and Irish)	128 [21.0]	89 [34.4]	39 [11.1]
South or South East Asian	53 [8.7]	11 [4.2]	42 [12.0]
Black British/Caribbean/African	10 [1.6]	3 [1.2]	7 [2.0]
Mixed or other background	58 [9.5]	27 [10.4]	31 [8.9]
Education attainment			
Higher education (e.g., degree and teaching)	247 [40.6]	106 [40.9]	141 [40.3]
High school/GCSE/A levels	203 [33.4]	55 [21.2]	148 [42.3]
Other/no qualifications	159 [26.1]	98 [37.8]	61 [17.4]
Employment			
Employed/self‐employed	212 [34.8]	91 [35.1]	121 [34.6]
Full‐time education	270 [44.3]	62 [24.0]	208 [59.4]
Unemployed/receipt of benefits	102[16.7]	84 [32.4]	18 [5.1]
Retired/other	24 [3.9]	21 [8.1]	3 [0.9]
Missing (other data provided)	1 [0.2]	1 [0.4]	0
Self‐report current/previous psychosis diagnosis^a^	259 [42.5]	259 [100.0]	0
Schizophrenia or paranoid schizophrenia	57 [9.4]	57 [22.0]	0
Schizoaffective	75 [12.3]	75 [29.0]	0
Schizophreniform	4 [0.7]	4 [1.5]	0
Delusional disorder	10 [1.6]	10 [3.9]	0
Depression with psychotic features	73 [12.0]	73 [28.2]	0
Bipolar with psychotic features	57 [9.4]	57 [22.0]	0
Brief psychotic disorder	15 [2.5]	15 [5.8]	0
Any other including psychotic experience	43 [7.1]	43 [16.6]	0
Past or current antipsychotic medication	140 [23.0]	138 [53.3]	2 [0.6]

*Note:* Percentages do not all sum to 100% due to rounding error.

^a^ Participants could select more than one diagnosis.

**TABLE 2:** Descriptive statistics for psychometric measures for entire sample, diagnosis versus no psychosis diagnosis subgroups and differences between the two subgroups.

	Entire sample			Psychosis diagnosis		No psychosis diagnosis		*p* ^a^
	*N*	Range	Mean (SD)	*N*	Mean (SD)	*N*	Mean (SD)	
CAPE‐42								
Positive symptoms	526	4–16	5.53 (2.57)	219	7.08 (3.08)	310	4.43 (1.30)	< 0.001
Hallucinations	532	5–20	9.91 (3.52)	218	11.86 (3.85)	314	8.55 (2.51)	< 0.001
Paranoia	531	7–35	10.58 (4.26)	219	12.79 (5.01)	312	9.03 (2.73)	< 0.001
Bizarre experiences	533	2–8	3.49 (1.62)	219	4.03 (1.84)	314	3.11 (1.33)	< 0.001
Grandiosity	531	2–8	3.10 (1.47)	219	3.64 (1.70)	312	2.72 (1.15)	< 0.001
Magical thinking								
Negative symptoms	532	4–16	8.43 (2.91)	219	9.56 (3.02)	313	7.63 (2.55)	< 0.001
Social withdrawal	533	3–12	5.74 (2.62)	219	6.89 (2.82)	314	4.93 (2.12)	< 0.001
Affect flattening	531	7–28	15.65 (5.08)	219	17.98 (5.00)	312	14.02 (4.46)	< 0.001
Avolition								
PAM‐R Insecure attachment styles								
Anxious	475	0–3	1.34 (0.77)	197	1.68 (0.74)	278	1.11 (0.70)	< 0.001
Avoidance	475	0–3	1.64 (0.74)	197	1.88 (0.72)	278	1.47 (0.71)	< 0.001
Disorganised	475	0–3	1.01 (0.81)	197	1.43 (0.81)	278	0.71 (0.67)	< 0.001
DES Dissociation subscales								
Amnesia	536	0–100	13.84 (19.46)	226	23.18 (23.91)	310	7.03 (11.36)	< 0.001
Depersonalisation/derealisation	536	0–100	19.69 (26.27)	226	34.77 (29.94)	310	9.23 (15.60)	< 0.001
Absorption	536	0–100	34.39 (25.68)	226	46.42 (27.50)	310	25.62 (20.20)	< 0.001
BBCS Negative Schemas								
Negative‐self	549	0–24	7.96 (5.92)	231	10.97 (6.12)	318	5.77 (4.69)	< 0.001
Negative‐other	549	0–24	8.76 (5.26)	231	10.73 (5.43)	318	7.33 (4.65)	< 0.001
BBTS Childhood Trauma: Abuse‐specific items								
Physical (two items)	501	2–6	2.59 (1.13)	204	2.94 (1.35)	297	2.36 (0.87)	< 0.001
Sexual (two items)	501	2–6	2.65 (1.04)	204	3.08 (1.27)	297	2.35 (0.72)	< 0.001
Emotional (one item)^b^								*χ* ^*2*^
Present	243	n/a	48.7%	137	n/a	106	n/a	< 0.001
Not present	256	n/a	51.3%	53	n/a	203	n/a	< 0.001

*Note:* Continuous variables are reported as mean and standard deviation (SD).

^a^ Non‐parametric Mann–Whitney *U* tests utilised as all continuous variables were non‐normally distributed. *χ* ^*2*^ chi‐square test used for binary emotional abuse variable.

^b^ Transformed to binary variable (present/not present).

### 4.2 Entire Sample Network Description

**FIGURE 1:**
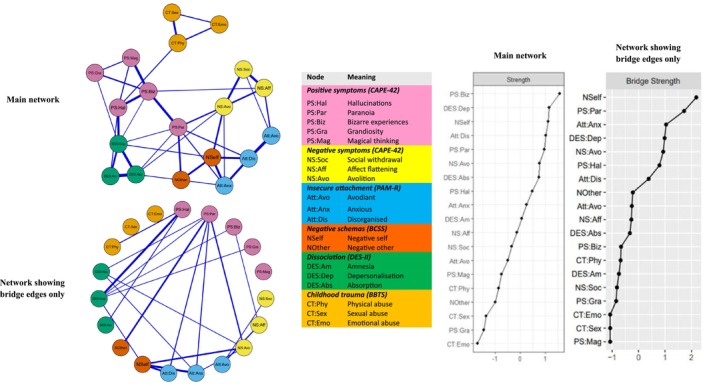
Graphical display of main network and network display of only the bridge edges. Corresponding strength centrality indices plots reported as *z* scores. Blue edges in the network graphs indicate positive unique correlations. The edge weight (thickness) is proportionate to the strength of the correlation. The main network graph shows all internode associations. The network graph displaying bridge edges only depicts on the edges between constructs, with intraconstruct associations set to zero.

Figure 1 shows the entire sample network in two forms and the corresponding centrality indices. The first (main) network graph shows all unique between‐node associations. The second, BEO, network graph shows only between‐construct edges, with intraconstruct associations set to zero.

Visual inspection of the main network revealed that nodes belonging to the same construct were clustered contiguously and were strongly associated. The centrality indices showed that bizarre experiences demonstrated the highest centrality, reflecting their high connectivity with other positive symptom domains. Centrality difference plots (see Figures S1 and S2) showed bizarre experiences to be significantly more central than the other positive symptom domains of magical thinking and grandiosity but not paranoia or hallucinations. Depersonalisation–derealisation was the most central dissociation dimension, showing strong connectivity with other dissociation domains and hallucinations. It was significantly more central than amnesia but not absorption. Negative‐self schemas represented the third most central node and were significantly more central than negative‐other schemas, reflecting their strong interconstruct connectivity and bridging connections with insecure attachment and avolition. Disorganised attachment had significantly higher centrality than avoidant attachment and anxious attachment. Avolition showed the highest ranked centrality of all negative symptoms and was significantly more central than social withdrawal but not negative affect.

The BEO centrality indices reflect only the strength of cross‐construct (bridging) node connectivity. Bizarre experiences showed considerably reduced centrality compared with the main network. Negative‐self schemas showed the highest bridging connectivity and were significantly more central than all other variables except paranoia. Paranoia showed significantly higher bridging connectivity than all other positive symptoms except hallucinations, reflecting its broad interconstruct connectivity. Anxious attachment showed the highest bridging connectivity of the insecure attachment domains, and this was significantly higher than avoidant but not disorganised attachment. Depersonalisation was the most central dissociative domain, showing significantly higher bridging connectivity than both amnesia and absorption, reflecting its strong connectivity with hallucinations. Avolition was again the most central negative symptom and showed significantly higher bridging connectivity than either negative affect or social withdrawal.

Correlation stability coefficients for the main and BEO networks were high (≥ 0.75) for strength centrality indices and edge weights (see Figures S3–S9 for results of strength centrality stability and edge‐weight accuracy tests). Therefore, the rank‐ordering of variables and differences in edge weights were considered reliable (Epskamp et al. 2018).

### 4.3 Psychosis Diagnosis and No Psychosis Diagnosis Subgroup Network Comparison

**FIGURE 2:**
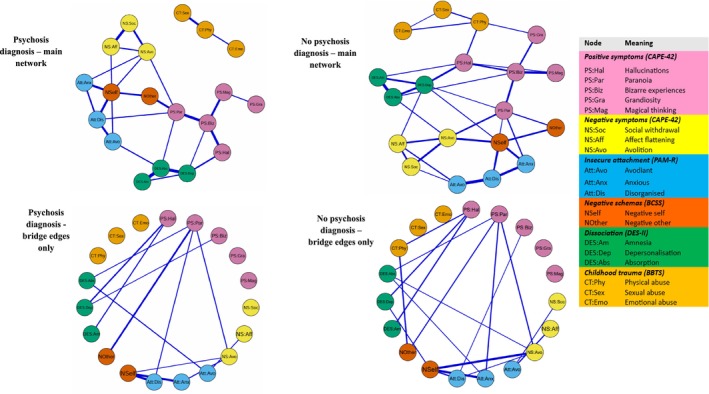
Graphical display of the psychosis diagnosis and no psychosis diagnosis main and ‘bridge edges only’ networks. The upper row of network graphs shows all internode associations. The lower row of network graphs shows only bridge edges—those between constructs, with intraconstruct associations set to zero.

**FIGURE 3:**
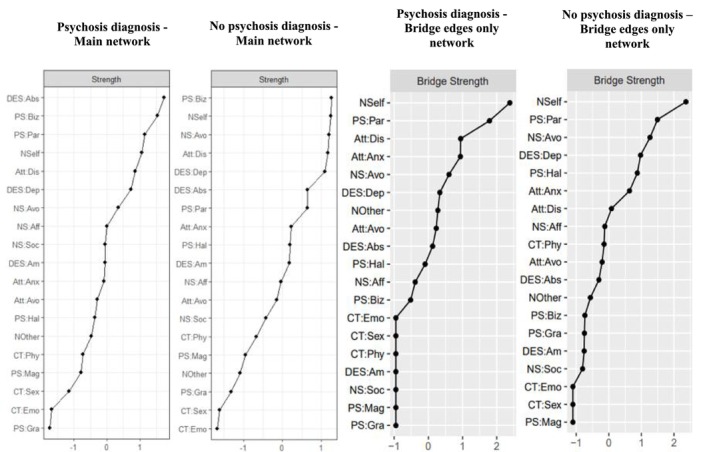
Psychosis diagnosis and no psychosis diagnosis subgroup main and bridge edges only (BEO) network strength centrality indices.

Figure 2 shows the psychosis diagnosis and no psychosis diagnosis main and BEO networks. Notably, the childhood trauma variables in the psychosis diagnosis network formed an isolated cluster, not uniquely associated with variables across constructs. The psychosis diagnosis sample also produced a sparser network with 27 non‐zero edges out of a possible 171 as opposed to 35 in the no‐diagnosis network. Centrality strength indices for the main and BEO networks are presented for the two subgroups in Figure 3.

The NCT showed no significant difference in network structure (M‐test = 0.17; *p =* 0.78). The overall strength of the edges in both networks was highly similar (5.33 vs. 5.51). This difference was not significant: S‐test = 0.18; *p =* 0.73. Significant edge weight differences were found for the connections between (i) avoidant attachment and absorption (*p =* 0.002), only present in the psychosis diagnosis network, and (ii) grandiosity and physical abuse (*p =* 0.017), only present in the no psychosis diagnosis network.

The rank order of the main network nodes, as depicted in Figure 3, showed considerable consistency, with bizarre experiences, negative‐self schemas and disorganised attachment all within the top five rankings. Noticeably, two insecure attachment domains (anxious and disorganised) were found within the five most central nodes in the psychosis BEO network, while none were in the top five rankings of the no psychosis diagnosis sample.

The NCT of edges identified in the BEO network graphs showed no significant differences. Adequate stability levels were observed for the psychosis diagnosis (edge = 0.67; strength = 0.60) and no psychosis diagnosis (edge = 0.67; strength = 0.59) networks.

## 5 Discussion

The aims of this study were to identify variables with the strongest network influence and consider their connections in relation to putative causal processes in psychosis symptoms. The study also aimed to assess whether network structures showed a statistically significant difference between subgroups of participants with or without a diagnosis of psychosis.

The finding that bizarre experiences, together with paranoia and hallucinations, demonstrated the highest strength centrality among positive symptoms within the main network suggests these variables combined had an influential role in positive symptom activation within the network. Cognitive models of delusions and hallucinations (e.g., Garety et al. 2001; Waters et al. 2012) propose a combination of factors such as source‐monitoring deficits, negative/threat‐based schemas, externalising attributions and negative affect contribute to the development of hallucinations and paranoid delusions. The centrality and strong connections between bizarre experiences (conceptually similar to delusions), paranoia (linked to threat‐based schemas) and hallucinations in the current network suggest the presence of shared and interacting processes underlying their connectivity. From a network‐theory perspective, identifying and disrupting the shared and interacting processes underlying the connectivity of these central variables should be a priority for psychological assessment and intervention.

Negative‐self schemas, paranoia and anxious attachment were shown, statistically, to be the most influential nodes within a constellation of strong cross‐construct connections including negative‐other schemas, disorganised attachment and avolition. Interestingly, the connection between paranoia and negative schemas occurred through negative‐*other* schemas, while the bridge between insecure attachment (anxious and disorganised) and negative schemas went through negative‐*self* schemas. These connections mirror findings that associations between paranoia and negative‐other, but not negative‐self schemas, tend to remain significant after controlling for confounders (Humphrey et al. 2021). Moreover, anxious and disorganised attachment both contain a core feature of negative‐self beliefs and are more strongly associated with negative‐self schemas than avoidant attachment (Bartholomew and Horowitz 1991). From a network‐theory perspective, this constellation suggests negative‐self and negative‐other schematic structures, together with paranoia and avolition are implicated in a set of self‐sustaining interactions indicative of mental ‘disorder’ (Borsboom 2017). Correspondingly, cognitive models of paranoid delusions highlight the combined impact of negative‐self/other beliefs leading to the perception of others as hostile and themselves as personally vulnerable (Freeman et al. 2002).

The finding that avolition and negative affect were statistically the most central negative symptom links to research suggesting that amotivation may be particularly influential in contributing to poor functioning in schizophrenia (Strauss et al. 2021; Foussias and Remington 2010). Negative affect showed high connectivity to other negative symptoms, while avolition showed unique cross‐construct associations with both negative‐self schemas and paranoia. One can intuit that negative beliefs about the self, combined with an elevated sense of threat, could reduce motivation and lead to reinforcing cycles of avoidance. These findings indicate that avolition may be usefully addressed alongside paranoia and negative‐self beliefs, which are already identified as an intervention priority in cognitive models of negative symptoms (Grant and Beck 2009).

Cognitive models of auditory hallucinations propose that dissociation plays a predisposing role (Berry et al. 2017). Depersonalisation–derealisation was statistically significantly more central than the other DES domains and was most strongly associated with hallucinations. Depersonalisation–derealisation is conceptualised as a ‘detachment’ form of dissociation (Brown 2006; Holmes et al. 2005). According to this conceptualisation, detachment functions at a neurobiological level to defend against the effects of extreme affect, thereby aiding functioning in high‐threat situations. It may be that neurobiological processes underlying detachment interact with processes such as source‐monitoring thought to underlie hallucinatory experiences (Bentall 1990). However, the role of ‘compartmentalisation’ forms of dissociation may also play an important role in hallucinations and was not adequately measured in the current study.

Regarding the study's fourth aim, the NCT found no significant difference in network structures between the subgroups based on psychosis/no psychosis diagnosis. This was despite the psychosis diagnosis group scoring significantly higher on all measurement variables. Therefore, it may be that networks containing the factors investigated in the current study show considerable structural continuity across the continuum of clinical severity in psychosis. It is noticeable, however, that within the psychosis diagnosis sample, childhood trauma variables ceased to be connected to other constructs within the network. It is likely that a combination of high rates of reported trauma within the psychosis diagnosis sample and limited response options on the measures of childhood trauma (1‐ to 3‐point Likert scale) provided a reduced spread of scores through which associations could be detected.

### 5.1 Limitations and Future Research

Limitations arise from the cross‐sectional design and use of self‐report survey data. Cross‐sectional data limit inferences about the causal nature of observed associations and the longitudinal stability of the network structures could not be examined, reducing the reliability of findings. Replicating the current network model to a new data set could increase confidence in the patterns of associations observed in the current study.

It is acknowledged that the lack of collateral information or clinician oversight regarding self‐reported psychosis diagnosis warrants caution when interpreting the reliability of these categories, and therefore, the null finding of structural differences between the psychosis and no psychosis diagnosis networks. Relatedly, the gender imbalance of males (72.5%) and the low numbers of people from Black British/Caribbean/African heritage warrant caution when considering the representativeness of the current sample and generalisability of findings. It is of note that people in the psychosis diagnosis sample in the current study were more likely to come from a ‘White other’ ethnic background while the broader literature suggests rates of psychosis in the United Kingdom tend to be elevated in people with Black Caribbean or African heritage (see meta‐analysis by Kirkbride et al. 2012).

Measurement limitations included the adapted abuse‐specific items used as measures of childhood trauma, which provided limited variation in scores and likely contributed to their reduced influence within the networks. Further, the focus on intrapsychic processes belied investigation of associations between psychological processes and real‐world functioning, restricting the ecological validity of these findings. No dedicated measure of negative affect was included in the current analyses which negated explicit investigation of its relationships to symptoms and proposed causal mechanisms. This is a significant drawback as affective difficulties have been highlighted as an important ‘pathway to psychosis’ (e.g., Kramer et al. 2014) and shown to mediate important relationships between factors such as insecure attachment and positive symptoms (Partridge et al. 2022). Additionally, all included variables related to psychopathology which negated exploration of factors that disrupt network connectivity and could aid recovery‐focused interventions.

Tentative conclusions have been drawn in the current study, based on a combination of node centrality and wider literature findings, regarding which variables may be considered treatment priorities. It is acknowledged that the use of cross‐sectional data and undirected networks, limits any inferences on the direction of effects and the targeting of central nodes necessarily resulting in reduced network connectivity or elevated therapeutic effects. Furthermore, an assumption of network analysis is that all relevant variables are included within the model, an assumption which like all studies, is certainly unmet in the current one.

For network analyses to realise more clinically useful models of psychotic experiences, they must examine causal mechanisms at the appropriate level of granularity (Borsboom 2017). Using data from experience sampling methods (ESM) within time‐lagged networks provides opportunities for exploring changes over time in interrelationships between momentary affective states, cognitions, behaviours and environmental factors, preceding psychotic experiences. Applications of these methods to psychosis research do exist (Klippel et al. 2018; Panayi et al. 2025), and similar repeated‐measures studies investigating the constructs explored in the current study could provide valuable insights into causal processes implicated in psychotic experiences.

### 5.2 Clinical Implications

The constellation of strong, cross‐construct associations between paranoia, negative schemas, anxious and disorganised attachment and avolition suggests these variables could inform clinical formulations of the interacting processes implicated in paranoid states. The centrality of paranoia, negative‐self schemas and anxious attachment suggests that addressing a combination of external and internal threat‐based thinking processes (e.g., negative schemas and automatic thoughts about self, others and the world) may weaken strong, reinforcing associations within the network and therefore be effective intervention targets. Further, this constellation of interactions consists of constructs with both maintenance and longitudinal elements, which supports the use of longitudinal maintenance formulations, as recommended in prominent psychosis treatment manuals (e.g., Morrison 2017). That said, given the considerably larger effect sizes than traditional CBT in reductions of persecutory delusions from the Feeling Safe Programme randomised controlled trial (Freeman et al. 2021), which predominantly focuses on interrupting ‘current’ maintenance processes such as reasoning biases, worry and safety‐seeking behaviours, intervention efforts may be most effectively targeted at the level of current behaviours and thinking processes.

Interventions aimed at reducing detachment‐type dissociation may be helpful in the management of hallucinations. Within the psychosis literature, targeting dissociation as a mediating factor between adverse experiences and psychosis symptoms, including auditory hallucinations, is recommended (e.g., Longden et al. 2020). A small‐sample case series study adapting a CBT intervention to augment techniques specifically targeting dissociation, such as low arousal strategies, distress tolerance and grounding techniques, showed promising findings with meaningful improvements on measures of emotional distress and perceived recovery from psychosis (Varese et al. 2021). Wider literature on targeting dissociative experiences proposes that developing clients' prediction of and control over dissociative experiences can help them remain in the present moment, reduce their reliance on avoidance, thereby reducing the need for dissociation (Zerubavel and Messman‐Moore 2015). Limited evidence to date provides tentative support for the effectiveness of mindfulness in reducing dissociation in adolescents (Sharma et al. 2016) and reducing the distress caused by hallucination‐like experiences within a small non‐clinical sample (Langer et al. 2010). These approaches warrant further, larger scale evaluation in respect of improving frequency and distress of dissociative experiences and hallucinations.

The finding that specific dimensions of multidimensional constructs showed unique associations with other constructs highlights the importance of using multidimensional measures within clinical assessments. Further research is required to develop measures with greater phenomenological and conceptual specificity and clearer theoretical links to the underlying psychological mechanisms they purport to measure. Bottom‐up, phenomenology‐driven research methodologies such as those employed in the development of a new ‘Felt Sense of Anomaly’ dissociation measure (Černis, Beierl, et al. 2021), could assist clinicians in gaining structured, yet granular quantitative measures to guide their practice and research.

## 6 Conclusion

This study provides a novel analysis of interrelationships between psychosis symptoms and implicated causal factors. Interventions aimed at disrupting connectivity between paranoia, negative schemas, insecure attachment behaviours and avolition are indicated. Amelioration of negative‐self schemas and paranoia may lead to the largest reductions in connectivity between these pathological processes. Interventions targeting detachment forms of dissociation may be most helpful in reducing hallucinations. Assessment of multidimensional aspects of psychosis‐related psychopathology and inclusion of maintenance and longitudinal elements within formulations are recommended. Longitudinal network analyses are required to assess the reliability of the current findings and explore causal associations.

## Funding

The authors have nothing to report.

## Conflicts of Interest

The authors declare no conflicts of interest.

## Supporting information


**Figure S1:** Entire sample main network strength centrality difference plot.
**Figure S2:** Entire sample bridge network strength centrality difference plot.
**Figure S3:** Strength centrality stability correlations for entire sample main network (0.75).
**Figure S4:** Strength centrality stability correlations for entire sample bridge network (0.75).
**Figure S5:** Overlapping confidence intervals for edge weights within entire sample networks (edge stability coefficient = 0.75).
**Figure S6:** Strength centrality stability correlations for psychosis diagnosis network (0.60).
**Figure S7:** Strength centrality stability correlations for no psychosis diagnosis network (0.59).
**Figure S8:** Overlapping confidence intervals for edge weights within psychosis diagnosis main and bridge networks (edge stability coefficient = 0.67).
**Figure S9:** Overlapping confidence intervals for edge weights within no psychosis diagnosis main and bridge networks (edge stability coefficient = 0.67).

## Data Availability

The data that support the findings of this study are available on request from the corresponding author. The data are not publicly available due to privacy or ethical restrictions.
