# Supplementary material for: Interrelationships Among Childhood Trauma, Insecure Attachment, Dissociation, Negative Schemas and Psychosis Symptoms: A Network Analysis
Source: Clin Psychol Psychother. 2025 Dec 12;32(6):e70187. doi: 10.1002/cpp.70187 (PMC12699288; doi:10.1002/cpp.70187)
Supplement: Supplementary file 1 — Figure S1: Entire sample main network strength centrality difference plot. Figure S2: Entire sample bridge network strength centrality difference plot. Figure S3: Strength centrality stability correlations for entire sample main network (0.75). Figure S4: Strength centrality stability correlations for entire sample bridge network (0.75). Figure S5: Overlapping confidence intervals for edge weights within entire sample networks (edge stability coefficient = 0.75). Figure S6: Strength centrality stability correlations for psychosis diagnosis network (0.60). Figure S7: Strength centrality stability correlations for no psychosis diagnosis network (0.59). Figure S8: Overlapping confidence intervals for edge weights within psychosis diagnosis main and bridge networks (edge stability coefficient = 0.67). Figure S9: Overlapping confidence intervals for edge weights within no psychosis diagnosis main and bridge networks (edge stability coefficient = 0.67). [file CPP-32-e70187-s001.docx]

**S1:** Description of stability and edge-weight accuracy

Centrality stability refers to the level of reliability that can be placed on the observed rank ordering centrality indices (nodes). Case-dropping subset bootstrap methods recommended by (Epskamp et al. 2018) were employed. This function re-estimated the networks utilising increasingly smaller subsets of the original sample, assessing the associated reductions in correlations between the original centrality indices and the subset centrality indices. Epskamp et al. advise that correlations between the original and subset indices of 0.7 and above indicate reliable (stable) rank orderings of indices, between 0.25 and 0.7 indicate moderate reliability, and below 0.25 indicate rank ordering of indices are not reliable.

Edge weight accuracy denotes the degree of confidence which can be placed in the observed ranking of the edge weights. To assess the accuracy of edge-weights within the networks, bootstrapped 95% confidence intervals (CIs) were calculated for each edge. Two edge weight confidence intervals that do not overlap are considered to show a significant difference in the strength (Epskamp, et al. 2018).

The stability of centrality indices and accuracy of edge weights were assessed using the R package ‘bootnet’, based on 1,000 bootstrapped samples.

**S2:** Centrality difference plots for entire sample main and bridge networks.

*Figure 1 supplementary: Entire sample main network strength centrality difference plot*


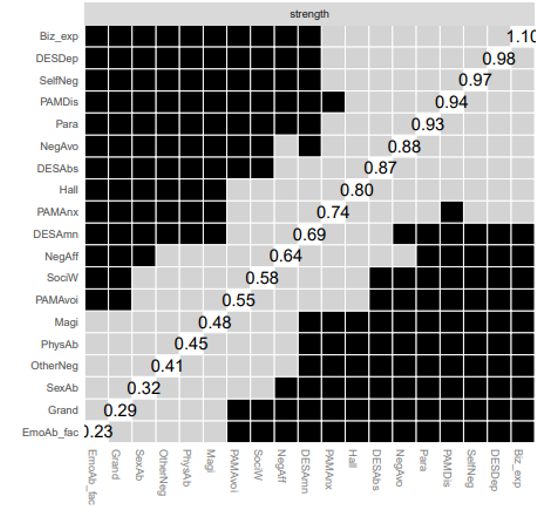


*Figure 2 supplementary: Entire sample bridge network strength centrality difference plot*


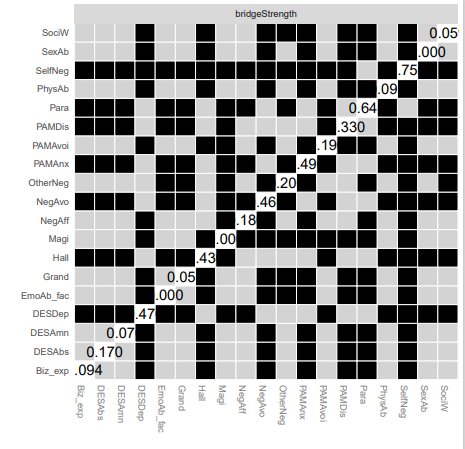


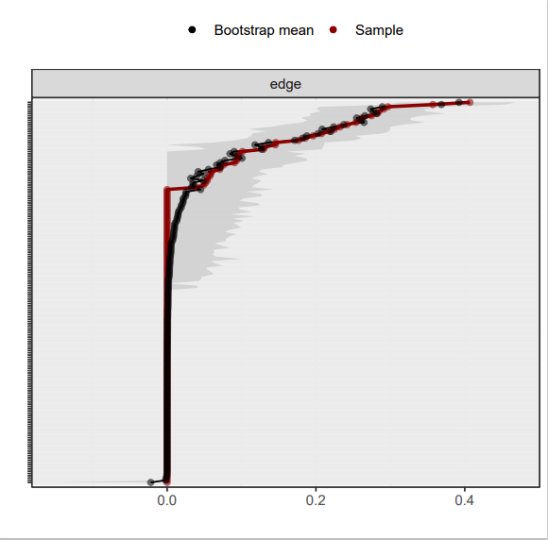

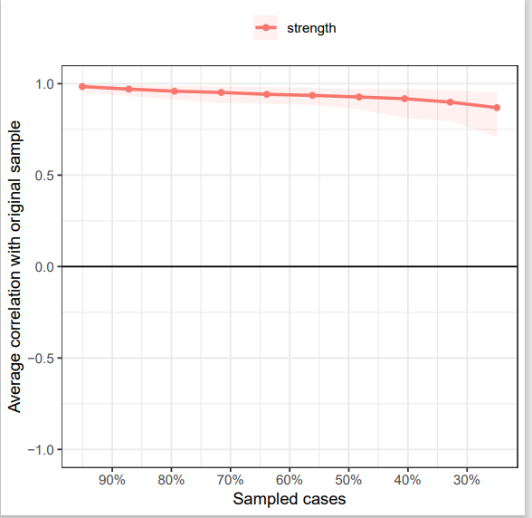

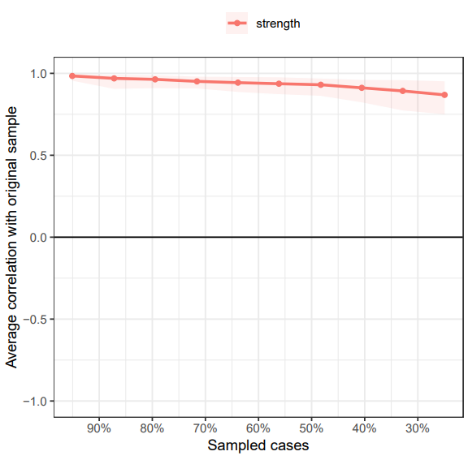
 **S3.** Figures 3-9 supplementary: Results from strength centrality stability and edge weight accuracy tests:

**
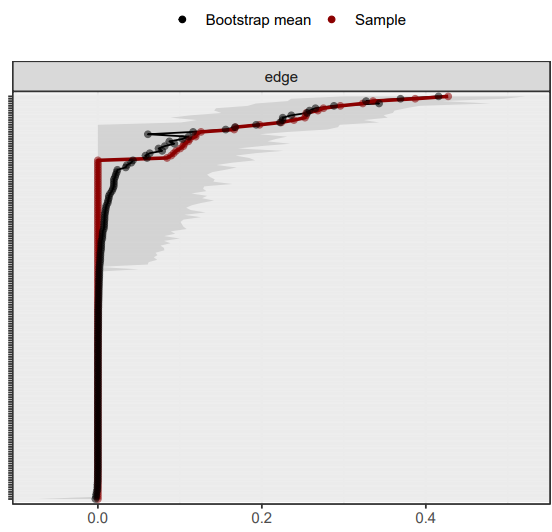
**
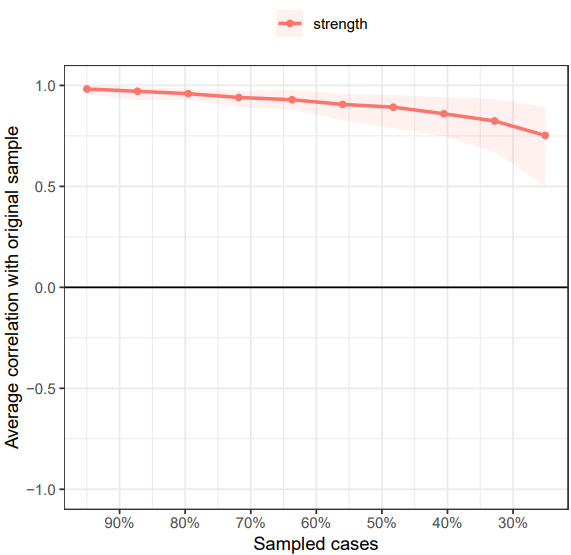

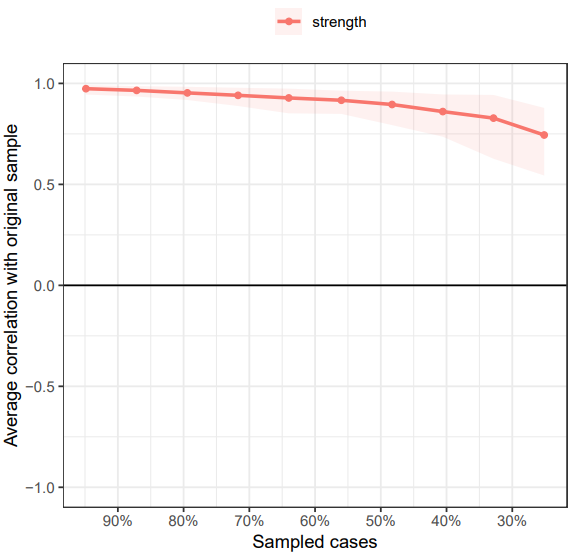


5

4

3

7

6

8


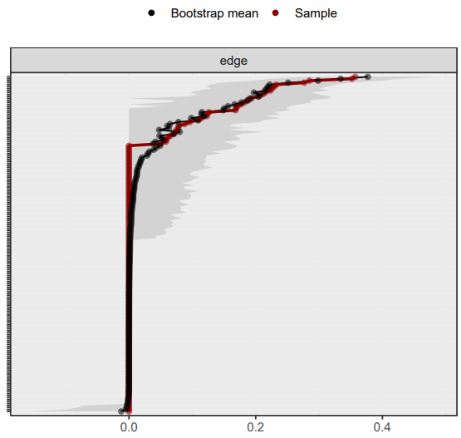


9

**Figure captions:**

3 = Strength centrality stability correlations for entire sample main network (.75)

4 = Strength centrality stability correlations for entire sample bridge network (.75)

5 = Overlapping confidence intervals for edge weights within entire sample networks (edge stability coefficient = .75)

6 = Strength centrality stability correlations for psychosis diagnosis network (.60)

7 = Strength centrality stability correlations for no psychosis diagnosis network (.59)

8 = Overlapping confidence intervals for edge weights within psychosis diagnosis main and bridge networks (edge stability coefficient = .67)

9 = Overlapping confidence intervals for edge weights within no psychosis diagnosis main and bridge networks (edge stability coefficient = .67)
